# Effect of carrageenan on the non-specific resistance of mice to injected syngeneic tumour cells, alone or in mixtures.

**DOI:** 10.1038/bjc.1979.47

**Published:** 1979-03

**Authors:** R. L. Wu, R. Kearney

## Abstract

The mechanisms of non-specific resistance to syngeneic methylcholanthrene-induced fibrosarcomas of mice were investigated. Results showed that a small tumour graft of 0.05 X 10(5) cells is greatly enhanced in growth when admixed with large numbers of cell fragments, killed cells or viable non-replicating cells. The enhancement of small tumour grafts in cell mixtures was found to be non-specific. Carrageenan, a known anti-macrophage agent, significantly increased tumour growth in normal mice. However, it did not enhance the increased tumour growth of 0.05 X 10(5) cells mixed with 10(6) viable, non-replicating mitomycin C-treated tumour cells. The latter observation indicates that carrageenan and admixed cells interfere with the same tumour-inhibitory mechanism and therefore cannot produce additive effects. The results give support to the concept of a non-specific macrophage "surveillance" system which appears crucial in controlling tumour growth, since it determines the establishment of small numbers of tumour cells while they can still be easily destroyed.


*Br. J. Cancer* (1979) **39**, 241

### EFFECT OF CARRAGEENAN ON THE NON-SPECIFIC RESISTANCE OF MICE TO INJECTED SYNGENEIC TUMOUR CELLS, ALONE OR IN MIXTURES

R. L. WU AND R. KEARNEY

*From the Department of Bacteriology, University of Sydney, Australia*

Received 16 October 1978 Accepted 22 November 1978

**Summary.—The mechanisms of non-specific resistance to syngeneic methylcholanthrene-induced fibrosarcomas of mice were investigated. Results showed that a small tumour graft of $0{\cdot}05 \times 10^5$ cells is greatly enhanced in growth when admixed with large numbers of cell fragments, killed cells or viable non-replicating cells. The enhancement of small tumour grafts in cell mixtures was found to be non-specific.**

**Carrageenan, a known anti-macrophage agent, significantly increased tumour growth in normal mice. However, it did not enhance the increased tumour growth of $0{\cdot}05 \times 10^5$ cells mixed with $10^6$ viable, non-replicating mitomycin C-treated tumour cells. The latter observation indicates that carrageenan and admixed cells interfere with the same tumour-inhibitory mechanism and therefore cannot produce additive effects. The results give support to the concept of a non-specific macrophage "surveillance" system which appears crucial in controlling tumour growth, since it determines the establishment of small numbers of tumour cells while they can still be easily destroyed.**

THE BEHAVIOUR and fate of small numbers of tumour cells have been a matter of considerable interest and speculation. Some tumours are capable of immediate growth by experimental inoculation or transplantation of a single cell (Furth & Kahn, 1937). Others fail to grow under similar circumstances and require relatively more cells before a tumour "take" occurs. Fisher & Fisher (1963) showed that an inoculum of a transplantable tumour, insufficient in numbers to produce growth, can start multiplication weeks later if some stimulus such as laparotomy, or an injection of tissue homogenate at the same implantation site is given. Révész (1958) found that in genetically compatible tumour–host systems, cells irreversibly damaged by heavy doses of radiation exert a profound enhancing influence on the growth of admixed viable cells. In subsequent experiments, Révész (1958) showed that the growth stimulation by irradiated cells of small numbers of viable cells failed to occur if the 2 populations were separated.

Recently, the concept of non-specific immune surveillance has been suggested as an important mechanism in the control of tumours (Alexander, 1976).

The following investigation was undertaken to determine whether carrageenan, a product of marine algae selectively cytotoxic for macrophages (Allison *et al.*, 1966; Schwartz & Leskowitz, 1969; Catanzaro *et al.*, 1971), would cause the establishment of small numbers of viable tumour cells which would not normally grow. Carrageenan is known to impair delayed hypersensitivity reactions *in vivo* (Bice *et al.*, 1971; Schwartz & Leskowitz, 1969) and is a potent immunosuppressant of antibody formation (Aschheim & Raffel,

Correspondence: R. Kearney, Department of Bacteriology, University of Sydney, Sydney, N.S.W. 2006, Australia.

1972; Thomson *et al.*, 1976). Keller (1976) and Thomson & Fowler (1977) have shown that carrageenan has an enhancing effect on the growth of relatively large tumour-cell inocula. Benjamini *et al.* (1977) reported that mitomycin C treatment of syngeneic tumour cells prevented replication of such cells, which could still be used to induce specific cell-mediated immunity. The following study involved mitomycin C-treated syngeneic fibrosarcoma cells as a source of non-replicating cells, to see whether they would cause the establishment of small numbers of tumour cells in mixtures and, if so, to determine whether such growth was enhanced by carrageenan.

### MATERIALS AND METHODS

*Animals.*—Male mice (8–10 weeks old) of the highly inbred CBA/H/WEHI strain were used. Their origin and maintenance have been discussed previously (Basten *et al.*, 1974).

*Tumours.*—Two syngeneic methylcholanthrene-induced fibrosarcomas designated H-1 and H-2 were used. Tumour-cell suspensions were prepared from solid tumours by pronase treatment (Kearney & Nelson, 1973). Viable tumour cells or mixtures in a total volume of 0·2 ml Dulbecco-modified Eagle's medium (DME), were injected s.c. along the midline of the abdominal wall. Tumour growth was expressed as the average tumour diameter (in units of 0·1 mm) by measuring, at daily intervals after the 4th day, the smallest and largest diameters with a Schnelltaster dial gauge as described previously (Kearney & Nelson, 1973), or by vernier calipers. The values given in the text are corrected for the average thickness of the uninjected-mouse skin-fold of the abdominal wall.

*Preparation of mitomycin C-treated tumour cells (MCT).*—Tumour cells obtained by incubation with 0·1% pronase in DME medium were washed $\times 3$ in DME medium with 20% foetal calf serum (FCS). Mitomycin C (MC; Kyowa Hakko Kogyo Co. Ltd, Japan) was dissolved in DME medium with 20% FCS and then added to the tumour cells at a concentration of 30 $\mu$g/$10^6$ H-1 tumour cells/ml medium. The cells were incubated at 37°C for 35 min and washed $\times 3$ in medium alone to remove free MC and FCS.

*Preparation of other inactivated cells.*—A single-cell suspension of untreated tumour cells was prepared as described, and suspended to a concentration of $2 \times 10^6$ cells in 0·2 ml of DME medium alone.

For heat treatment, this suspension was incubated at 56°C for 35 min. This treatment destroyed the viability of the tumour cells, as indicated by the trypan-blue exclusion test.

For freeze-thawing, the suspension was immersed in liquid $N_2$ and thawed under running tapwater. The procedure was repeated $\times 3$. Viability of the cells was totally destroyed as assessed by trypan-blue exclusion.

*Latex particles.*—Calibration latex (Coulter Electronics, Hertfordshire) of diameter 2·03 $\mu$m was washed $\times 3$ by centrifugation, in medium alone, before use.

*Preparation of mixtures of inactivated cells or particles with tumour cells.*—A single-cell suspension of untreated H-1 tumour cells containing $0{\cdot}1 \times 10^5$ cells/0·2 ml was prepared. This suspension was mixed *in vitro* with suspensions of MC-treated cells, heat-killed cells, frozen-thawed cells or latex particles containing the equivalent of $2 \times 10^6$ tumour cells per 0·2 ml. For this purpose, it was calculated that latex particles were $\frac{1}{6}$ the diameter of the average tumour cell and that therefore an equivalent volume to $2 \times 10^6$ cells would be provided by $\sim 2 \times 10^8$ latex particles.

These mixtures in a volume of 0·2 ml were immediately injected s.c. into the midline of the abdominal wall of normal mice. Therefore, each animal received $0{\cdot}05 \times 10^5$ live H-1 cells mixed with an equivalent of $10^6$ inactivated cells, or $10^8$ latex particles.

*Carrageenan.*—Lambda carrageenan (Marine Colloids Inc., Springfield, New Jersey) was dissolved in boiling physiological saline at a concentration of 0·5 mg/0·2 ml, and then stored at −20°C until required. The method of carrageenan pretreatment was as follows: animals were given 0·5 mg of carrageenan, injected i.p., on each of 3 days before the test procedure, *i.e.*, before the inoculation of viable cells or admixtures; on the day of the experiment, a final dose of 0·5 mg was administered, about 1 h before the tumour-cell inoculation; thus a total of 2 mg was administered in 4 divided doses.

### RESULTS

### *Admixture of* 0·05 × $10^5$ *H*-1 *cells with* $10^6$ *MC-treated tumour cells*

Fig. 1 and Tables I, II and III show that, whereas a graft of 0·05 × $10^5$ H-1 cells alone produces a tumour in a small minority of normal mice, the same number of cells, mixed with $10^6$ viable non-replicating MCT cells, will produce a tumour in all normal animals. The resultant tumours grow at a rate comparable to a dose of 0·5 × $10^5$-H-1 cells given alone (Fig. 1). The enhancement of a low dose of H-1 tumour cells is also seen when these cells are admixed with $10^6$ MC-treated H-2 tumour cells. As seen in Fig. 1, the growth of 0·05 × $10^5$ H-1 cells mixed with either $10^6$ MCT–H-1 or $10^6$ MCT–H-2 cells is very similar.

FIG. 1—Effect of admixture of $10^6$ MCT H-1 or MCT H-2 with 0·05 × $10^5$ H-1 tumour cells inoculated s.c.: ○, 0·05 × $10^5$ H-1 alone; ■, $10^6$ MCT H-1 alone; ■—■, 0·05 × $10^5$ H-1 + $10^6$ MCT H-1; □, $10^6$ MCT H-2 alone; □---□, 0·05 × $10^5$ H-1 + $10^6$ MCT H-2; ●—●, 0·5 × $10^5$ H-1 alone.

TABLE I.—*Effect of admixture of mitomycin C-treated tumour cells (MCT) to untreated tumour cells*

| Number of live untreated H-1 tumour cells (× $10^5$) | $10^6$ MCT cells added | Tumour incidence at Day 15 |
|---|---|---|
| 0·05 | — | 3/7 |
| 0·5 | — | 7/7 |
| — | H-1 | 0/8 |
| — | H-2 | 0/8 |
| 0·05 | H-1 | 8/8 |
| 0·05 | H-2 | 8/8 |

FIG. 2—Effect of carrageenan on growth of admixed MCT H-1 and untreated H-1 tumour cells ■—■, $10^6$ MCT H-1 alone; ○—○, 0·05 × $10^5$ H-1 alone; ■---■, 0·05 × $10^5$H-1 + $10^6$ MCT H-1; △---△, 0·05 × $10^5$ H-1 + carrageenan; □—□, 0·05 × $10^5$ H-1 + $10^6$ MCT H-1 + carrageenan; ●—●, 0·5 × $10^5$ H-1 alone; ▲---▲, 0·5 × $10^5$ H-1 + carrageenan.

### *Effect of carrageenan pretreatment on the growth of H-*1 *tumour cells either alone or in mixtures*

The effect of carrageenan pretreatment on the growth of a small graft (0·05 × $10^5$ H-1 cells) mixed with $10^6$ MCT–H-1 cells is shown in Fig. 2.

Although carrageenan pretreatment significantly enhanced the growth of 0·5 × $10^5$ cells and caused a 100% take of 0·05 × $10^5$ H-1 cells, it did not affect the growth rate of 0·05 × $10^5$ H-1 cells in mixtures.

### *Effect of admixture of killed cells*

In Table II it can be seen that mixture with $10^6$ heat-killed or freeze-thawed cells also produced enhancement of $0{\cdot}05 \times 10^5$ H-1 cells. However, the resultant enhancement is not as pronounced as that seen when MCT cells are used (Fig. 3). However, if latex particles are mixed with such a graft, tumour growth is totally prevented. This also occurred with a large, optimal dose of tumour cells, so that it is probable that the latex particles were toxic for the cells. A violent inflammatory reaction was seen to occur at the site of injection of latex particles, followed by considerable local ulceration.

TABLE II.—*Effect of admixture of killed cells and latex on tumour growth*

| Inoculum live H-1 tumour cells ($\times 10^5$) | No. of admixed cells or latex particles | Tumour incidence at Day 20 |
|---|---|---|
| 0·05 | — | 4/8 |
| 0·5 | — | 8/8 |
| 0·05 | $10^6$ MCT H-1 | 8/8 |
| 0·05 | $10^6$ Heat-killed H-1 | 8/8 |
| 0·05 | $10^6$ Frozen-thawed H-1 | 7/8 |
| 0·05 | $10^8$ Latex | 0/8 |
| 0·5 | $10^6$ Frozen-thawed H-1 | 8/8 |
| 0·5 | $10^8$ Latex | 1/8 |

FIG. 3—Effect of admixture of either MCT H-1 cells or dead cells with $0{\cdot}05 \times 10^5$ H-1 cells: ●—●, $0{\cdot}05 \times 10^5$ H-1 cells + $10^6$ MCT H-1 cells; △—△, $0{\cdot}05 \times 10^5$ H-1 cells + $10^6$ frozen-thawed H-1 cells; ▲—▲, $0{\cdot}05 \times 10^5$ H-1 cells + $10^6$ heat-killed H-1 cells.

No growth (not shown) of either $10^6$ MCT H-1 or $0{\cdot}05 \times 10^5$ H-1 cells (as in Figs. 1 and 2).

*Effect of inoculating MC-treated tumour cells at a site distant from a small live graft*

A dose of $0{\cdot}05 \times 10^5$ untreated H-1 cells was inoculated s.c. into normal mice. At the same time, $10^6$ MCT–H-1 or MCT–H-2 cells were inoculated i.p. Table III shows that MCT cells separated from the small graft did not promote tumour enhancement as seen with mixtures.

TABLE III.—*Effect of mitomycin C-treated tumour cells (MCT) given i.p., on the growth of a small tumour graft given s.c.*

| Cells given i.p. | Cells given s.c. | Tumour incidence at Day 20 |
|---|---|---|
| — | $10^6$ MCT H-1 | 0/7 |
| — | $10^6$ MCT H-1 + $0{\cdot}05 \times 10^5$ H-1 | 8/8 |
| — | $0{\cdot}05 \times 10^5$ H-1 | 1/7 |
| $10^6$ MCT H-1 | $0{\cdot}05 \times 10^5$ H-1 | 2/8 |
| — | $10^6$ MCT H-2 | 0/7 |
| — | $10^6$ MCT H-2 + $0{\cdot}05 \times 10^5$ H-1 | 8/8 |
| $10^6$ MCT H-2 | $0{\cdot}05 \times 10^5$ H-1 | 1/8 |

### DISCUSSION

These experiments demonstrate that a small tumour graft is greatly enhanced in growth when mixed with large numbers of inactivated tumour cells. The same effect results whether or not cells of the same antigenicity are used (H-1 and H-2 tumours do not cross-react antigenically; Wu & Kearney, submitted for publication) and is not dependent on the viability of the added cells. However, the 2 cell populations must be mixed, as a simultaneous injection of inactivated cells and a small graft at 2 different sites does not produce the effect.

Using lethally irradiated cells, Révész (1958) observed the same effect. His results were confirmed and extended by Toda *et al.* (1967), Yatvin *et al.* (1970) and Hewitt *et al.* (1973). The explanation which best fits existing data is that the admixed tumour cells interfere with a local, non-specific clearance mechanism which can normally remove small numbers of injected cells.

The existence of a macrophage-mediated tumour-inhibitory mechanism was indicated by the enhancement of tumour growth by the administration of carrageenan. This observation has been previously reported for both chemically induced (Keller, 1976; Nelson & Nelson, 1978) and virally induced (Lotzova & Richie, 1977) tumours.

It is unlikely that the enhancement is due to a direct stimulatory effect of carrageenan on tumour growth, since carrageenan has been shown to be toxic for tumour cells *in vitro* (Pollack & Nelson, 1973), although products from damaged macrophages may have an enhancing effect (Keller, 1976). Carrageenan has many biological effects (Di Rosa, 1972) including anticoagulant activity (Anderson & Duncan, 1965) and inhibition of complement (Borsos *et al.*, 1965), all of which may affect tumour growth. However, the most likely explanation for the enhancement is that it is due to inhibition of macrophage function. Further evidence for this belief is found in the report of Keller (1976), who showed that poly-vinyl-pyridine-N-oxide (PVNO) could prevent the enhancement of tumour growth by both carrageenan and silica. PVNO selectively reverses the toxic effects of these agents on macrophages.

If carrageenan is enhancing tumour growth in normal animals by crippling macrophage function, 2 possible mechanisms should be considered:

Firstly, it is conceivable that carrageenan may have prevented the development of specific immunity which normally restrains tumour growth. This is unlikely, since tumour growth has already begun before specific immunity would be expected to have an effect. Also, in view of the lack of effect of carrageenan on the induction and expression of specific immunity by MCT cells (Wu & Kearney, submitted for publication), it is unlikely that such a mechanism is responsible for the observed enhancement.

A second, more plausible, explanation is that carrageenan pretreatment interferes with a non-specific, macrophage-mediated, tumour-inhibitory system which is important in clearing small numbers of tumour cells in animals without specific immunity. This mechanism may mediate a primitive surveillance system which can recognize and dispose of small numbers of neoplasitc cells, thus preventing the development of small grafts but only able to reduce the growth potential of larger grafts.

In the context of the present experiments, it could be postulated that admixed inactivated tumour cells could protect small numbers of live cells by providing macrophages with an overwhelming load to handle. Thus the potent cells could escape destruction, while the macrophages were preoccupied with removing harmless, inactivated or dead cells. The observation that carrageenan neither increased nor decreased the effect may be an indication that both carrageenan and admixed cells interfere with the same tumour-inhibitory mechanism and therefore cannot produce additive effects.

In summary these findings, together with the observation that carrageenan greatly enhances tumour growth, lend some support to the concept that a non-specific, macrophage-mediated "surveillance" system may be as important as specific immunity in determining the growth of syngeneic tumours. Because of the weakness of specific H-1 tumour immunity (Wu & Kearney, submitted for publication), it could be argued that, in this instance, the non-specific mechanism is crucial in controlling tumour growth, since it prevents the establishment of small numbers of tumour cells while they are still capable of being easily controlled. However, this concept does not negate the importance of specific immune mechanisms, which may be decisive in limiting the growth of an established and growing tumour.

This investigation was supported by grants from the University of Sydney Cancer Research Committee, New South Wales State Cancer Council and the Medical Research Committee of the University of Sydney. Miss R. L. Wu was the recipient of an AMSA-Lilly Research Scholarship.

### REFERENCES

ALEXANDER, P. (1976) The functions of the macrophage in malignant disease. *Ann. Rev. Med.*, **27**, 207.

ALLISON, A. C., HARRINGTON, J. S. & BIRBECK, M. (1966) An examination of the cytotoxic effects of silica on macrophages. *J. Exp. Med.*, **124**, 141.

ANDERSON, W. & DUNCAN, J. G. C. (1965) The anti-coagulant activity of carrageenan. *J. Pharm. Pharmacol.*, **17**, 647.

ASCHHEIM, L. & RAFFEL, S. (1972) The immunodepressant effect of carrageenan. *J. Reticuloendothel. Soc.*, **11**, 253.

BASTEN, A., MILLER, J. F. A. P., SPRENT, J. & CHEERS, C. (1974) Cell-to-cell interaction in the immune response. X. T-cell dependent suppression in tolerant mice. *J. Exp. Med.*, **140**, 199.

BENJAMINI, E., FONG, S., ERICKSON, C., LEUNG, C. Y., RENNICK, D. & SCIBIENSKI, R. J. (1977) Immunity to lymphoid tumours induced in syngeneic mice by immunization with mitomycin C-treated cells. *J. Immunol.*, **118**, 685.

BICE, D. E., SCHWARTZ, H. J. & LAKE, W. W. (1971) Effect of carrageenan on the establishment of delayed hypersensitivity. *Int. Arch. Allergy Appl. Immunol.*, **41**, 628.

BOROS, T., HERBERT, J. R. & CRISLER, C. (1965) The interaction between carrageenan and the first component of complement. *J. Immunol.*, **94**, 662.

CATANZARO, P. J., SCHWARTZ, J. H. & GRAHAM, R. C. (1971) Spectrum and possible mechanism of carrageenan cytotoxicity. *Am. J. Pathol.*, **64**, 387.

DI ROSA, J. (1972) Biological properties of carrageenan. *J. Pharm. Pharmacol.*, **24**, 89.

FISHER, B. & FISHER, E. R. (1963) Local factors affecting tumour growth. I. Effect of tissue homogenates. *Cancer Res.*, **23**, 1651.

FURTH, J. & KAHN, M. C. (1937) The transmission of leukaemia of mice with a single cell. *Am. J. Cancer*, **31**, 276.

HEWITT, H. B., BLAKE, E. & PORTER, E. H. (1973) The effect of lethally irradiated cells on the transplantability of murine tumours. *Br. J. Cancer*, **28**, 123.

KEARNEY, R. & NELSON, D. S. (1973) Concomitant immunity to syngeneic methylcholanthrene-induced tumours in mice. Occurrence and specificity of concomitant immunity. *Aust. J. Exp. Biol. Med. Sci.*, **51**, 723.

KELLER, R. (1976) Promotion of tumour growth *in vivo* by antimacrophage agents. *J. Natl Cancer Inst.*, **57**, 1355.

LOTZOVA, E. & RICHIE, E. R. (1977) Promotion of incidence of adenovirus type II transplantable tumours by carrageenan, a specific anti-macrophage agent. *J. Natl Cancer Inst.*, **58**, 1171.

NELSON, M. & NELSON, D. S. (1978) Macrophages and resistance to tumours. Influence of agents affecting macrophage and delayed-type hypersensitivity on resistance to tumours inducing concomitant immunity. *Aust. J. Exp. Biol. Med. Sci.*, **56**, 211.

POLLACK, S. & NELSON, K. (1973) Effects of carrageenan and high serum dilutions on synergistic cytotoxicity to tumour cells. *J. Immunol.*, **110**, 1440.

RÉVÉSZ, L. (1958) The effect of lethally damaged tumour cells upon the development of admixed viable cells. *J. Natl Cancer Inst.*, **20**, 1157.

SCHWARTZ, H. J. & LESKOWITZ, S. (1969) The effect of carrageenan on delayed hypersensitivity reactions. *J. Immunol.*, **103**, 87.

THOMSON, A. W., WILSON, A. R., CRUICKSHANK, W. J. & HORNE, C. H. W. (1976) Evaluation of carrageenan as an immunosuppressive agent and mediator of intravascular coagulation. *Biomedicine*, **23**, 102.

THOMSON, A. W. & FOWLER, E. W. (1977) Potentiation of tumour growth by carrageenan, *Transplantation*, **24**, 397.

TODA, J. K., YATVIN, M. B. & CLIFTON, K. H. (1967) Source of stimulation of tumour inocula by lethally irradiated cells. *Proc. Soc. Exp. Biol. Med.*, **125**, 241.

YATVIN, M. B., CROISE, D. T. & CLIFTON, K. H. (1970) Studies on the mechanism of the stimulatory effect of lethally irradiated cells on tumour inocula. *Proc. Soc. Exp. Biol. Med.*, **125**, 241.


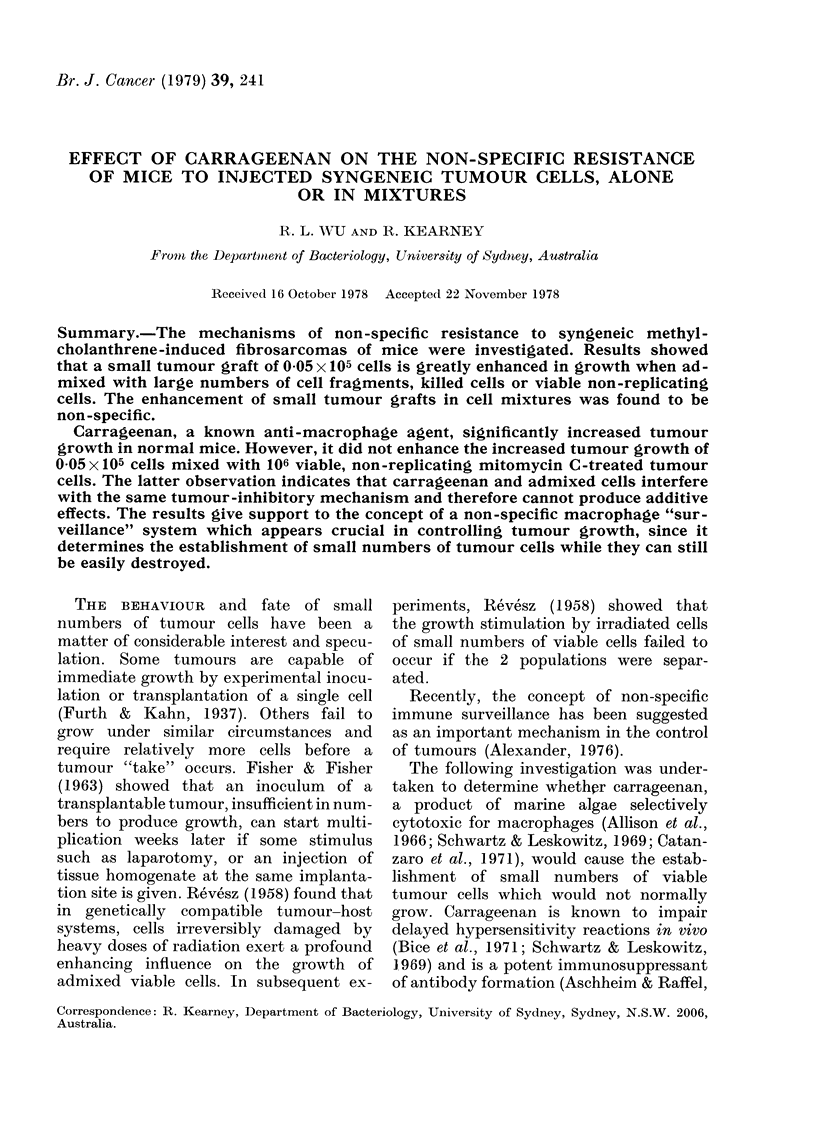

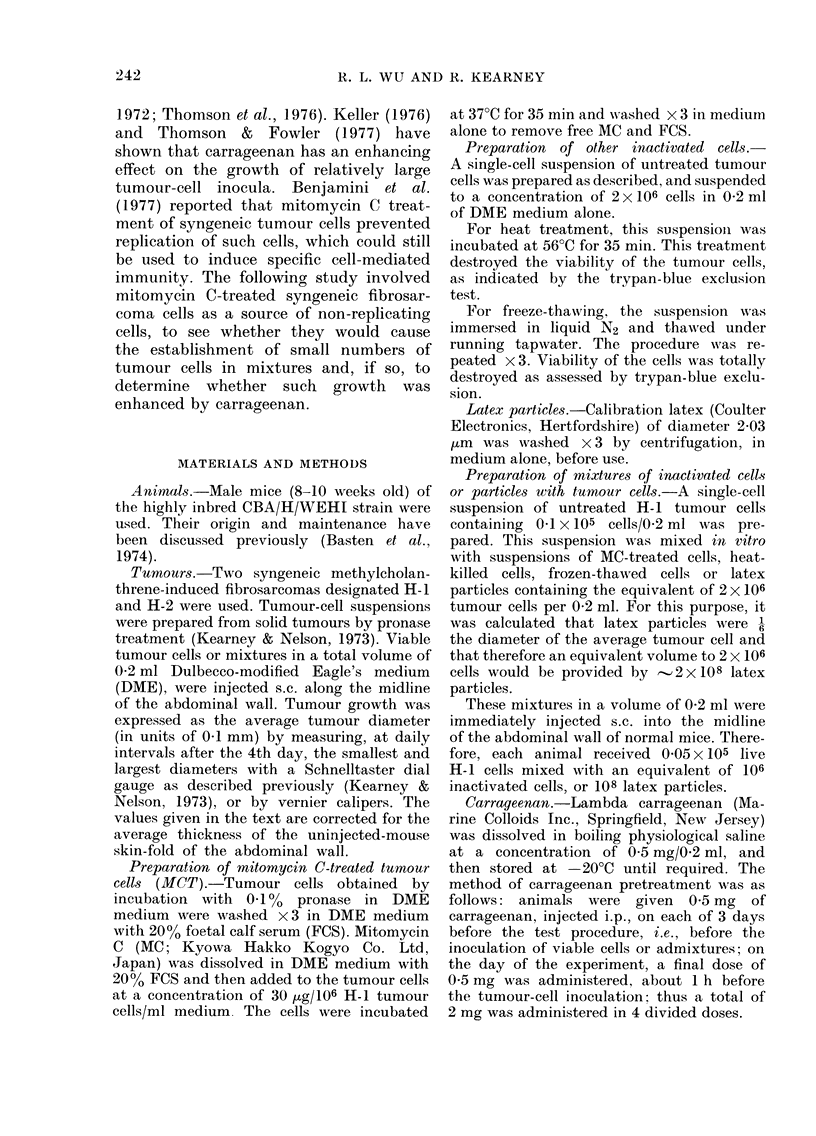

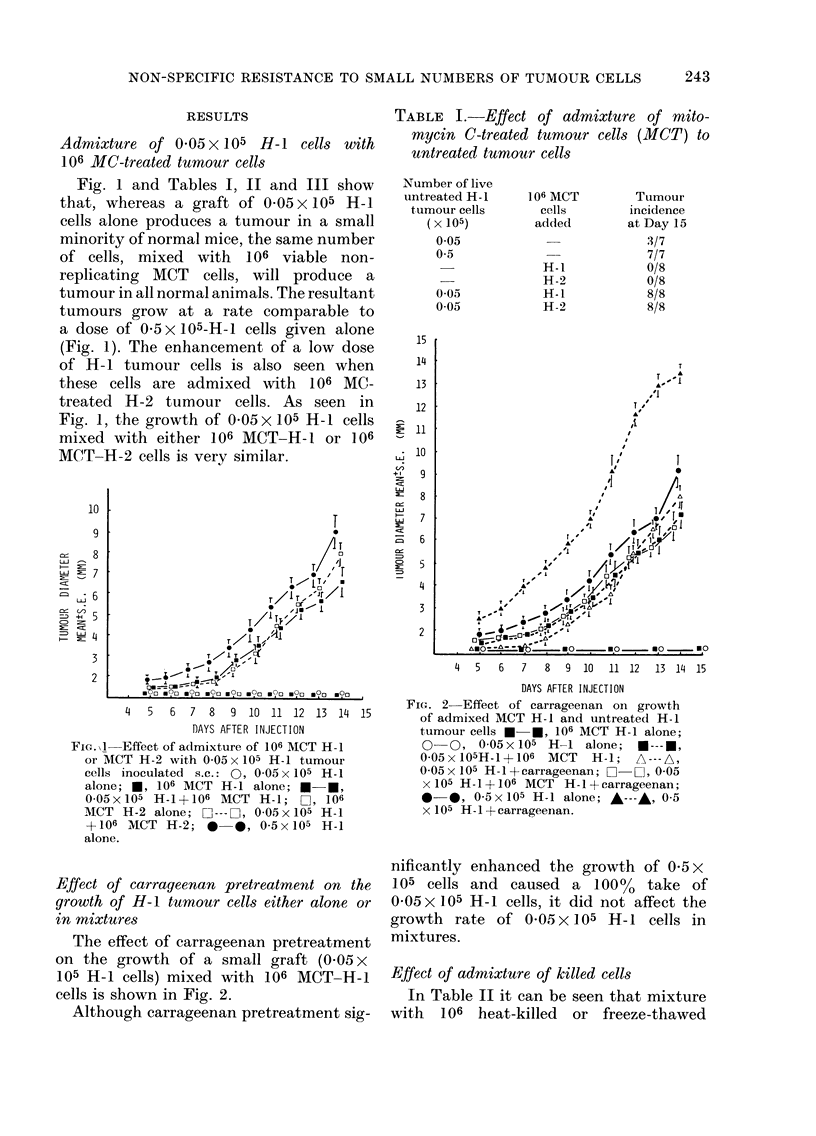

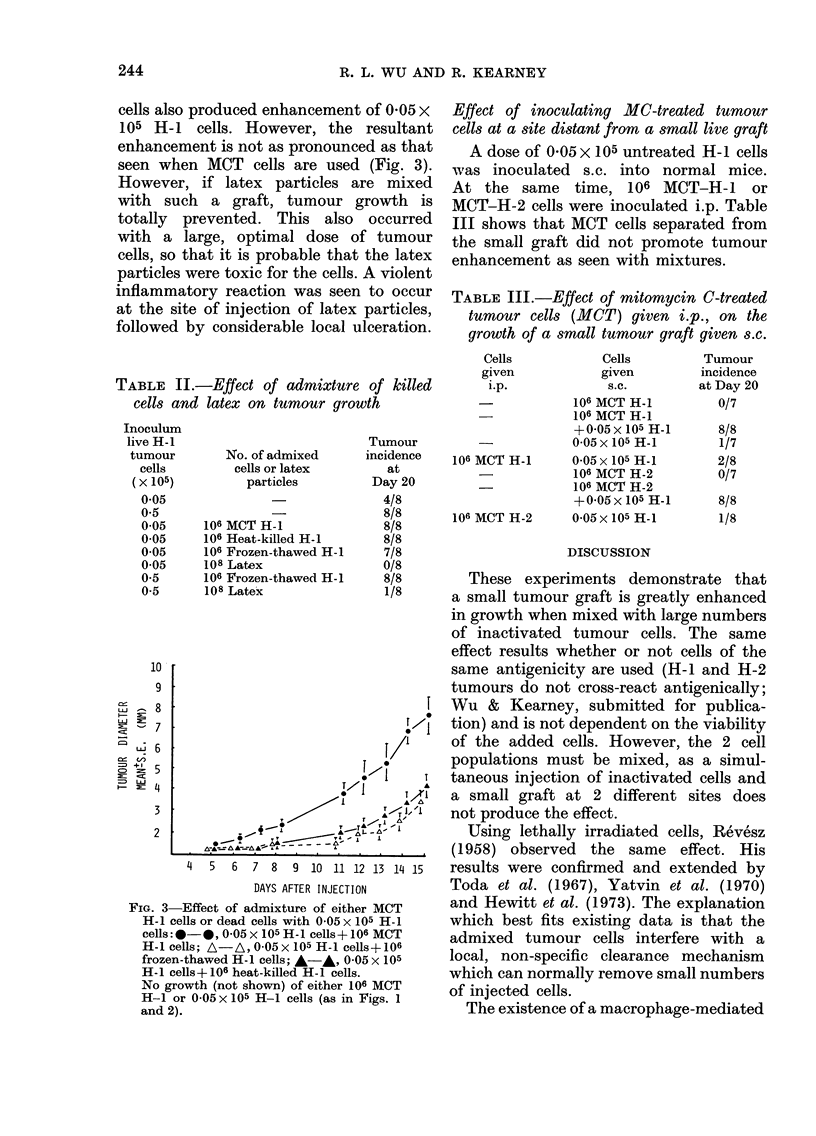

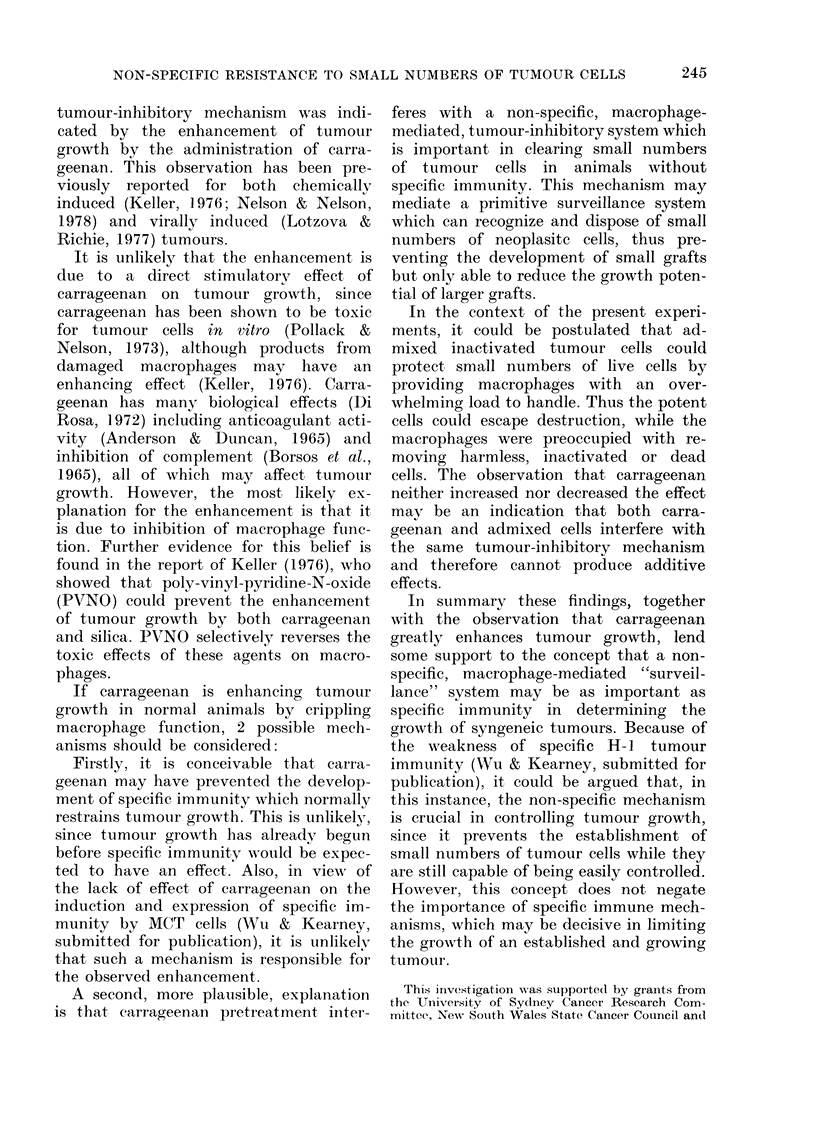

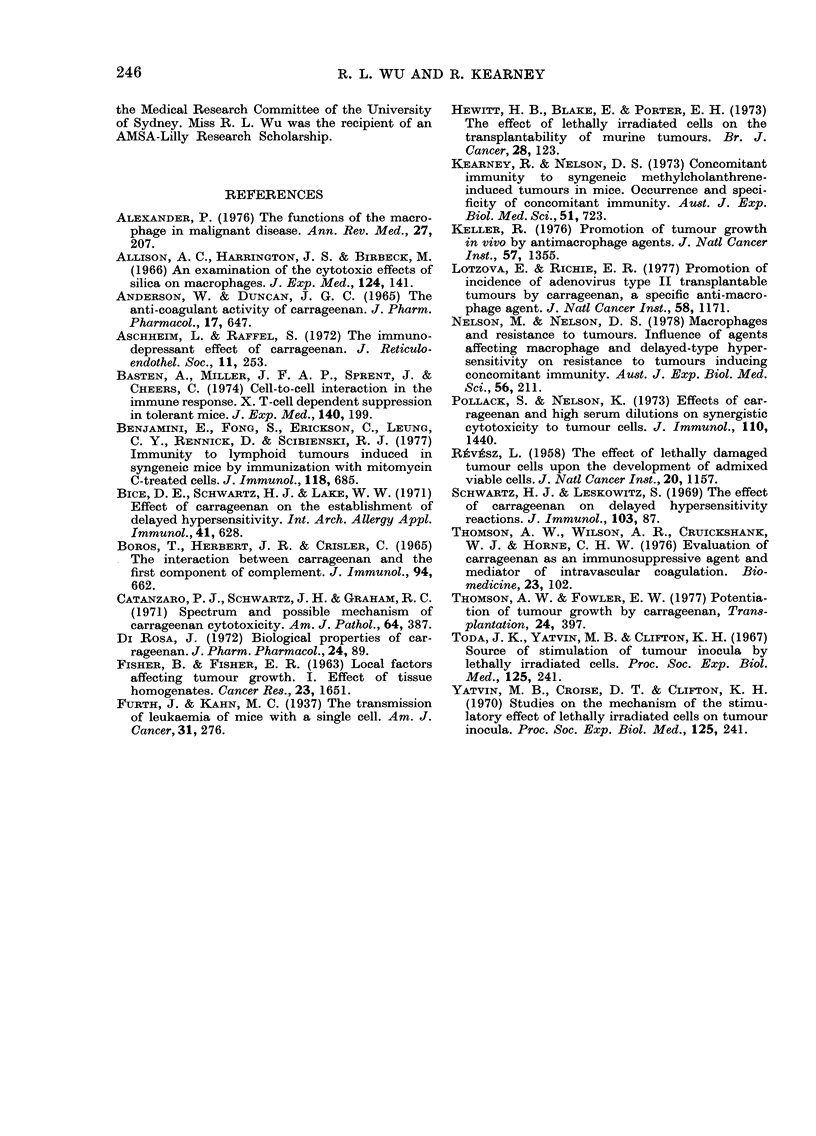


## References

[OCR_00701] Alexander P. (1976). The functions of the macrophage in malignant disease.. Annu Rev Med.

[OCR_00706] Allison A. C., Harington J. S., Birbeck M. (1966). An examination of the cytotoxic effects of silica on macrophages.. J Exp Med.

[OCR_00711] Anderson W., Duncan J. G. (1965). The anticoagulant activity of carrageenan.. J Pharm Pharmacol.

[OCR_00716] Aschheim L., Raffel S. (1972). The immunodepressant effect of carrageenin.. J Reticuloendothel Soc.

[OCR_00740] BORSOS T., RAPP H. J., CRISLER C. (1965). THE INTERACTION BETWEEN CARRAGEENAN AND THE FIRST COMPONENT OF COMPLEMENT.. J Immunol.

[OCR_00721] Basten A., Miller J. F., Sprent J., Cheers C. (1974). Cell-to-cell interaction in the immune response. X. T-cell-dependent suppression in tolerant mice.. J Exp Med.

[OCR_00727] Benjamini E., Fong S., Erickson C., Leung C. Y., Rennick D., Scibienski R. J. (1977). Immunity to lymphoid tumors in syngeneic mice by immunization with mitomycin C-treated cells.. J Immunol.

[OCR_00734] Bice D., Schwartz H. J., Lake W. W., Salvaggio J. (1971). The effect of carrageenan on the establishment of delayed hypersensitivity.. Int Arch Allergy Appl Immunol.

[OCR_00746] Catanzaro P. J., Schwartz H. J., Graham R. C. (1971). Spectrum and possible mechanism of carrageenan cytotoxicity.. Am J Pathol.

[OCR_00750] Di Rosa M. (1972). Biological properties of carrageenan.. J Pharm Pharmacol.

[OCR_00754] FISHER B., FISHER E. R. (1963). LOCAL FACTORS AFFECTING TUMOR GROWTH. I. EFFECT OF TISSUE HOMOGENATES.. Cancer Res.

[OCR_00764] Hewitt H. B., Blake E., Proter E. H. (1973). The effect of lethally irradiated cells on the transplantability of murine tumours.. Br J Cancer.

[OCR_00770] Kearney R., Nelson D. S. (1973). Concomitant immunity to syngeneic methylcholanthrene-induced tumours in mice. Occurrence and specificity of concomitant immunity.. Aust J Exp Biol Med Sci.

[OCR_00777] Keller R. (1976). Promotion of tumor growth in vivo by antimacrophage agents.. J Natl Cancer Inst.

[OCR_00782] Lotzová E., Richie E. R. (1977). Promotion of incidence of adenovirus type 12 transplantable tumors by carrageenan, a specific antimacrophage agent.. J Natl Cancer Inst.

[OCR_00788] Nelson M., Nelson D. S. (1978). Macrophages and resistance to tumours: influence of agents affecting macrophages and delayed-type hypersensitivity on resistance to tumours inducing concomitant immunity.. Aust J Exp Biol Med Sci.

[OCR_00796] Pollack S., Nelson K. (1973). Effects of carrageenan and high serum dilution on synergistic cytotoxicity to tumor cells.. J Immunol.

[OCR_00802] REVESZ L. (1958). Effect of lethally damaged tumor cells upon the development of admixed viable cells.. J Natl Cancer Inst.

[OCR_00807] Schwartz H. J., Leskowitz S. (1969). The effect of carrageenan on delayed hypersensitivity reactions.. J Immunol.

[OCR_00819] Thomson A. W., Fowler E. F. (1977). Potentiation of tumour growth by carrageenan.. Transplantation.

[OCR_00812] Thomson A. W., Wilson A. R., Cruickshank W. J., Horne C. H. (1976). Evaluation of carrageenan as an immunosuppressive agent and mediator of intravascular coagulation.. Biomedicine.

[OCR_00830] Toda J. K., Yatvin M. B., Clifton K. H. (1967). Source of stimulation of tumor inocula by lethally irradiated cells.. Proc Soc Exp Biol Med.

[OCR_00824] Toda J. K., Yatvin M. B., Clifton K. H. (1967). Source of stimulation of tumor inocula by lethally irradiated cells.. Proc Soc Exp Biol Med.

